# GPU Acceleration of Melody Accurate Matching in Query-by-Humming

**DOI:** 10.1155/2014/614193

**Published:** 2014-02-12

**Authors:** Limin Xiao, Yao Zheng, Wenqi Tang, Guangchao Yao, Li Ruan

**Affiliations:** ^1^State Key Laboratory of Software Development Environment, Beihang University, Beijing 100191, China; ^2^School of Computer Science and Engineering, Beihang University, Beijing 100191, China; ^3^Aviation Institute, Beijing 101121, China

## Abstract

With the increasing scale of the melody database, the query-by-humming system faces the trade-offs between response speed and retrieval accuracy. Melody accurate matching is the key factor to restrict the response speed. In this paper, we present a GPU acceleration method for melody accurate matching, in order to improve the response speed without reducing retrieval accuracy. The method develops two parallel strategies (intra-task parallelism and inter-task parallelism) to obtain accelerated effects. The efficiency of our method is validated through extensive experiments. Evaluation results show that our single GPU implementation achieves 20x to 40x speedup ratio, when compared to a typical general purpose CPU's execution time.

## 1. Introduction

With the development of information technology, music retrieval technology has been widely applied. Query-by-humming (QBH) is an important application for music retrieval, where users can hum a melody to retrieve the song [1–3]. Different from the traditional music retrieval engine, which searches the song based on the description of the music such as the singer or the song name, QBH retrieves the song based on the content. Music retrieval is becoming more natural, simple, and user-friendly with the advancement of QBH. Thus, QBH will give broader application prospects for music retrieval [4–6].

Typical QBH systems consist of three modules, including feature extraction, melody database, and melody matching [7]. The feature extraction module includes pitch extract and note segmentation. Pitch extract means that the acoustic input is first put into frames; then we obtain the pitch frequencies from the frames; finally we use (1) to transform them into the representation of semitone. Note segmentation means that the obtained pitch sequence is cut into different notes. The melody database module is responsible for the management and index MIDI melody information. Consider

(1) $$\begin{array}{c} \text{semitone}=12*\log_{2}\left(\frac{\text{freq}}{440}\right)+69. \end{array}$$

The core of QBH is the melody matching between the humming melody and the melody database. From the view of practical application, melody matching algorithms can be divided into fast match and accurate match [8, 9]. Fast match mainly removed the less likely candidates from the melody database, in order to reduce the computation of accurate match and improve the system's response time; accurate match then calculated the more exact similarities between the humming melody and the candidate melodies, so as to obtain the final list of similar songs. Hence, we can see that the characteristics of accurate match are a large amount of calculation and precise calculations.

There has to be a trade-off between response speed and accuracy, if the QBH system wants to improve the retrieval accuracy within acceptable times. However, with the increasing scale of the melody database, the size of candidate melodies is also growing, which results in an extended response time and poor user experience. The usual method for improving the response speed is to reduce the size of the candidate melodies, but this may cause low retrieval accuracy [10].

Therefore, many efforts are made to develop methods and techniques that execute the melody matching in high performance platforms, allowing the production of accurate results in a shorter time. Graphic Processing Unit (GPU) is one of the recent trends in high performance platforms, which has demonstrated significant speedups for many scientific applications [11, 12]. Researchers also have studied the melody matching on GPUs [13, 14]. However, existing works choose to implement a coarse-grained parallelization of the melody matching algorithm, where multiple problem instances are simply replicated onto each multiprocessor of a GPU.

In this paper, by analyzing the existing accurate matching algorithms, we propose a GPU-based parallelization for the accurate matching algorithm. Our proposed method develops two parallel strategies (intratask parallelism and intertask parallelism). By taking full advantage of multicomputing units of GPU, our method can greatly decrease the computation time of the accurate match algorithm and improve the system's response speed without reducing the retrieval accuracy.

The rest of this paper is organized as follows.  Section 2 summarizes the melody accurate matching. In Section 3, an outline of the CUDA-based GPU computing platform is given.  Section 4 describes the details of our parallelization strategy for melody accurate matching using CUDA. Experimental results are analyzed in Section 5. The last section concludes the whole paper and points out some future works.

## 2. Melody Accurate Matching in QBH

### 2.1. Problem Definition

In a QBH system, the humming input is firstly put into a pitch tracker frame by frame and then the output pitch sequence is converted to a semitone scale according to (1). The MIDI main melody extracted from the multitrack melody is put into a note sequence.

**Definition 1**

The humming pitch sequence at semitone scale of length *m* is denoted by *Q* = (*q*_1_, *q*_2_, …, *q*_*m*_), where *q*_*i*_ represents the pitch corresponding to the *i*th frame of humming.

**Definition 2**

The MIDI note sequence matched with *Q* is denoted by *P* = {(*p*_1_, *d*_1_), (*p*_2_, *d*_2_), …, (*p*_*n*_, *d*_*n*_)}, where *p*_*i*_ and *d*_*i*_ represent the pitch and duration of the *i*th note, respectively.

**Definition 3**

The distance function between the humming sequence and the MIDI note sequence is denoted by dist⁡(*q*_*i*_, *p*_*j*_), where *q*_*i*_ and *p*_*j*_ represent the pitch of humming sequence and MIDI melody.

Therefore, based on the above definitions, the problem of melody accurate matching could be expressed, given humming sequence *Q*, MIDI note sequence *P*, and distance function, how to calculate the minimum distance between the two sequences.

### 2.2. Melody Accurate Matching Algorithms

In practical humming applications, different people have different vocal ranges, resulting in tonal inconsistency between humming melody and standard melody but tonal change consistency; similarly, different people have different singing speed, resulting in singing speed ratio inconsistency between the two melodies. Thus, there exist speed change and pitch offset between humming melody and standard MIDI melody. How to evaluate the speed change and pitch offset before accurate matching is the difficulty of melody accurate matching algorithms. Existing matching algorithms, including linear scaling (LS) [15], recursive alignment (RA) [9], and dynamic time warping (DTW) [16], take different approaches to resolve the above difficulty and improve retrieval accuracy.

Linear scaling (LS) is a simple, intuitive, and effective melody matching algorithm. LS chooses different scaling factors to stretch or compress the pitch contour of the humming melody to more accurately match the MIDI melody. The deficiency of LS is also quite obvious: local mismatch may deteriorate the global matching; the selection of suitable stretching coefficient or pitch offset is difficult; the length of humming melody may affect the retrieval accuracy.

Recursive alignment (RA) uses recursive linear scaling match to explore the optimal matching results. Although it is derived from the LS, this method overcomes the disadvantage of using single stretch factor or pitch offset throughout the entire melody matching. Hence, RA is capable of capturing long distance information in human singing. The drawbacks of this approach are matching the finer, higher time complexity; the segmentation fragments based on midpoint, and the MIDI melodies do not necessarily satisfy the linear relationship.

Dynamic time warping (DTW) is one of the most effective approaches to melody accurate matching, which is a frame-based dynamic programming algorithm. In DTW, the candidate note sequences should be expanded to frame sequences. Compared to LS and RA, DTW has higher accuracy but higher complexity. The details of DTW-based melody accuracy matching are described in following section.

## 3. GPU Computing with CUDA

Over the past few years, GPU has gained significant popularity as a powerful tool for high performance computing. With the development of general programming toolkits, such as Compute Unified Device Architecture (CUDA), GPU becomes a general-purpose shared-memory many-core computing platform and plays important roles in applications such as computer vision, search, and sorting [17, 18].

CUDA is an extension of C/C++ which enables users to write scalable multithreaded programs for CUDA-enabled GPUs [19]. In the CUDA programming model, an application consists of a sequential host program, which can execute parallel programs known as kernels on GPUs. A kernel is executed using potentially large number of parallel threads. Thus, GPU achieves massive parallelism through executing a large number of lightweight threads concurrently. These threads are organized in thread blocks and grids of thread blocks. Each thread runs the same sequential program and has a thread ID within its thread block. The hierarchical organization into blocks and grids has implications for thread communication and synchronization. Threads within a thread block can communicate through a per-block shared memory and may synchronize using barriers. However, threads located in different blocks cannot communicate or synchronize directly. The memory model in CUDA has five types of memory: each thread has private local memory; each thread block has shared memory visible to all threads of the block; all threads have access to the same global memory; there are also two additional read-only memories accessible by all threads: the constant, and texture memory.

Thread creation, scheduling, and management are performed entirely in hardware. In order to manage large number of threads, the GPU employs the SIMT (Single Instruction Multiple Thread) architecture [20] in which the threads of a block are executed in groups of 32 called warps. The warp can greatly improve performance by having threads in a warp execute the same code path and access memory in nearby addresses.

## 4. GPU-Parallel Melody Accurate Matching

As described in Section 2.2, DTW is an efficient melody matching algorithm; however, the time complexity is *O*(*mt*). This is the motivation for our research, hoping that we can accelerate DTW computation using GPU without reducing the accuracy.

### 4.1. Melody Accurate Matching Based on DTW

The melody accurate matching based on DTW is outlined in Algorithm 1. Suppose that the query pitch sequence is denoted by *Q* = (*q*_1_, *q*_2_, …, *q*_*m*_), and the note sequence is denoted by *P* = {(*p*_1_, *d*_1_), (*p*_2_, *d*_2_), …, (*p*_*n*_, *d*_*n*_)}. Then we can construct a *m* × *t* DTW matrix *D* according to (2). *D*(*i*, *j*) is the minimum distance starting from the left-most side (*i* = 1) of the matrix to the current position (*i*, *j*). Consider

(2) $$\begin{array}{c} D\left(i,j\right)=\mathrm{dist}\left(q_{i},p_{j}^{\prime }\right)+\min\{\begin{array}{cc} D\left(i-2,j-1\right) & \\ D\left(i-1,j-1\right) & \\ D\left(i-1,j-2\right). & \end{array} \end{array}$$

The corresponding boundary conditions for the above recursion can be expressed as

(3) $$\begin{array}{c} D\left(i,1\right)=\infty ,\;i=2,\ldots ,m\\ D\left(1,j\right)=\mathrm{dist}\left(1,j\right),\;j=1,\ldots ,t\\ D\left(i,0\right)=\infty ,\;i=1,\ldots ,m\\ D\left(0,j\right)=\infty ,\;j=1,\ldots ,t\\ D\left(0,0\right)=0. \end{array}$$

The cost of the optimal DTW path is defined as

(4) $$\begin{array}{c} \underset{j=1\;\text{to}\;t}{\min}D\left(m,j\right). \end{array}$$

After finding the optimizing *j*, the optimal DTW path can be obtained by back tracking.

As previously described, tempo variation and pitch transposition should be considered before the accurate matching.

For most users, tempo variation is attributed to linear variation. Researchers apply the LS algorithm to the humming pitch sequence before comparing it to the candidate melody. The recurrent relation shows that the optimal path exists only when the humming input is within half to twice the size of the candidate melody. Hence, the humming sequence can be scaled several times, ranging from 0.5 to 2 times the original length and compared to the candidate melody in order to achieve the best scaling factor.

For pitch transposition, a heuristic method can be applied that shifts the entire humming pitch sequence to a suitable position in order to generate the minimum DTW distance.

### 4.2. Parallelization Strategy for Melody Accurate Matching

In this section, our proposed parallelization strategies for melody accurate matching based on DTW are explained. We investigate two approaches for parallelizing the melody accurate matching using CUDA: intra-task parallelization and inter-task parallelization. Intra-task parallelization indicates that each task is assigned to one thread block and all threads in the thread block cooperate to perform the task in parallel. Inter-task parallelization indicates that each task is assigned to exactly one thread and all threads in a thread block perform the tasks in parallel.

#### 4.2.1. Intra-Task Parallelization

**Figure 1:**
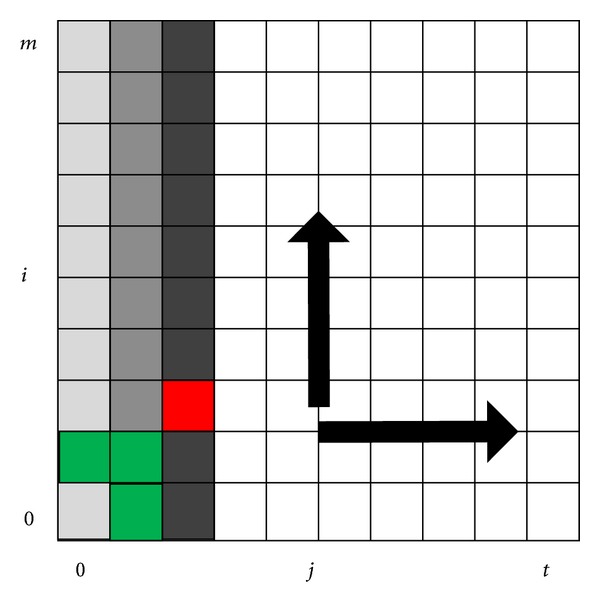
Column-wise fashion for computing matrix *D*.

As described in the above section, the DTW algorithm has quadratic time complexity that limits its usefulness. The purpose of computation during the DTW algorithm is to fill the matrix *D*, which can be easily implemented as a simple two nested loops. According to (2), a given *D*(*i*, *j*) can be computed only if *D*(*i* − 2, *j* − 1),  *D*(*i* − 1, *j* − 1), and *D*(*i* − 1, *j* − 2) have already been computed. For instance, as shown in Figure 1, *D*(2, 2) (red cell locates) depends on *D*(0,1), *D*(1,1), and *D*(1,0) (green cells locate). This indicates that all elements in column two are computable simultaneously if the elements in column zero and column one have already been computed. Thus, every entry of the same column is computable and elements within the column can be computed in parallel.

Based on this idea, we present an efficient parallel implementation for calculating matrix *D*, as shown in Algorithm 2. The input to the algorithm is two pitch sequences. The output of the algorithm is matrix *D*. Lines 3–5 are the core of our parallel algorithm, which calculates each element of a column in parallel. After calculating all elements of each column, synchronization operation is performed to ensure that all processors have finished current computation (line 6).

We can easily determine the time complexity of Algorithm 2. Assume that there are *p* processors for computing simultaneously. Based on Algorithm 2, the total execution time can be expressed as

(5) $$\begin{array}{c} T\left(m,t\right)=\sum_{j=2}^{t}\left\lceil \frac{m-1}{p}\right\rceil =O\left(\frac{1}{p}*mt\right). \end{array}$$

#### 4.2.2. Inter-Task Parallelization

As described in above the section, inter-task parallelization indicates that we compute in parallel all the scores of a humming query with every piece of melody in the candidate melody set. For instance, if the candidate set contains *N* pieces of melodies, we can launch *N* threads executing Algorithm 2 in parallel. The main challenges are the optimization of resource allocations and memory operations.

We store the humming pitch sequence in texture memory, which is the cached, fast memory and shared among all threads. Because of the increasing scale of the melody database, the candidate melody set usually contains too many pieces of melodies to be stored in any of the cached memories (texture, constant and shared memory). Therefore, we store the candidate melody set in the global memory. To gain maximum bandwidth and best performance, all threads in a half-warp should access the note sequences in global memory in a coalesced pattern. In order to adopt the coalesced pattern, every note sequence is stored in an array of double-float, a CUDA structure containing two 32-bit floats storing the pitch and duration of each note. Then we organize all note sequences in memory as a one-dimensional array, such that its first *N* entries correspond to the first note of each sequence; the next *N* entries correspond to the second note of each sequence, and so forth.

Each thread performs the computations on its own matrix *D*. In order to minimize the amount of required memory, we only store the current column and forward two columns of each matrix in shared memory. Finally, in order to allow simultaneous read/write operations by the active threads, we store the matrices using the same strategy as the note sequences.

### 4.3. Implementation with CUDA

Based on the above parallelization strategies, we present an acceleration method for melody accurate matching, as shown in Algorithm 3. Specifically, intra-task parallelization is performed in a thread block; inter-task parallelization is performed among thread blocks.

## 5. Performance Evaluation

In order to evaluate the performance of our GPU-parallel melody accurate matching algorithm, several experiments were carried out. We first introduce the singing corpus used in this study. Then, we compare the performance and accuracy between our parallel implementation and the existing sequential implementation.

### 5.1. Evaluation Environment

Evaluation environment is shown in Table 1. We use an AMD AthlonII X4 3.2 GHz for CPU, NVIDIA GTX285 with 240 SPs (Stream processor), and GTX480 with 480 SPs for GPUs.

To evaluate our proposed acceleration method, we used the publicly available MIREX (music information retrieval evaluation exchange) QBSH corpus [21], which has been used for the evaluation of QBSH for many times. The corpus includes 48 MIDI files and 4431 singing or humming clips. Each clip has duration of 8 s, with an 8 kHz sampling rate and 8 bit resolution. The frame size is 256 and the overlap is 0, resulting in a pitch rate of 8000/256 = 31.25. Hence, an 8 s singing clip is converted to a pitch vector of 31.25∗8 = 250 elements in semitones. We add 10000 MIDI files to MIREX corpus to compose the MIDI database. These MIDI files are collected from MIDI archives on the internet.

### 5.2. Performance Results

We introduce the speedup ratio to evaluate the performance of GPU-parallel melody accurate matching algorithm. Top-M hit rate and mean reciprocal rank (MRR) are used to evaluate the accuracy of our proposed algorithm. MRR is a standard metrics in MIREX, which can be denoted as

(6) $$\begin{array}{c} \text{MRR}=\frac{1}{n}\sum_{i=1}^{n}\frac{1}{{\mathrm{rank}}_{i}}, \end{array}$$

where rank⁡_*i*_ means the rank of the correct song for the *i*th query.

**Figure 2:**
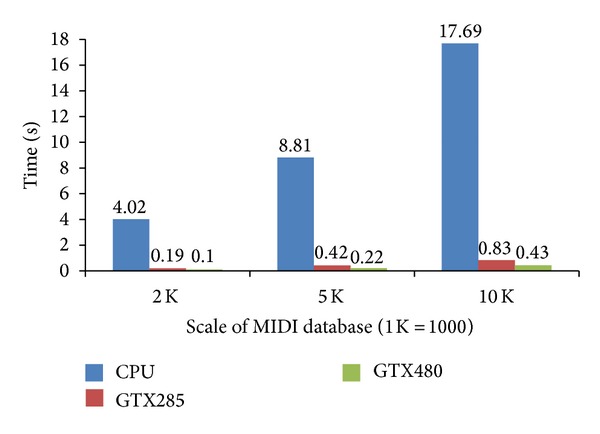
Comparison of execution times with different scale of MIDI database.

Figure 2 shows the execution times of different hardware settings with respect to the varying scale of MIDI database. We observe that the performance is sensitive to the problem size and hardware settings. The results indicate that the more cores are on GPU, the greater the performance is improved.

**Figure 3:**
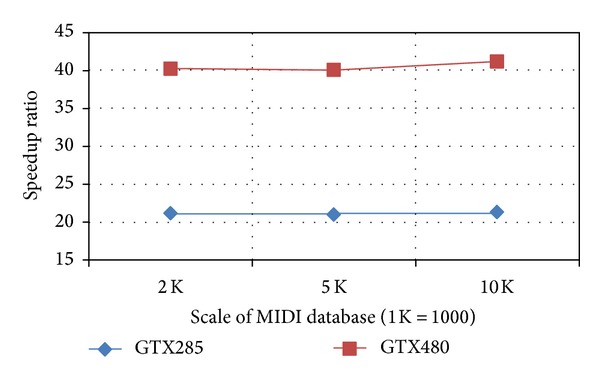
Speedup ratio obtained with the usage of GPUs.

As shown in Figure 3, the speedup ratio over CPU serial version ranges from 20 to 40 on GPUs. The results also indicate that the fluctuation of speedup ratio is very small as the scale of MIDI database increases.

To verify whether the accelerated strategy affects the retrieval accuracy, we evaluate the accuracy of our proposed parallel algorithm simultaneously. We selected 829 singing clips from the corpus to test the performance. As shown in Table 2, our proposed parallel algorithm almost does not affect the retrieval accuracy.

## 6. Conclusion and Future Work

This paper presents a GPU-parallel melody accurate matching method for query-by-humming. The method uses two parallelization strategies (intra-task parallelization and inter-task parallelization) to accelerate melody matching. The experimental results show that our proposed method can obtain 20x to 40x speedups without reducing retrieval accuracy.

For future work, we will further optimize the parallel program to improve the performance. Second, we will perform exhaustive comparison with other QBH acceleration methods.

## Figures and Tables

**Algorithm 1:**
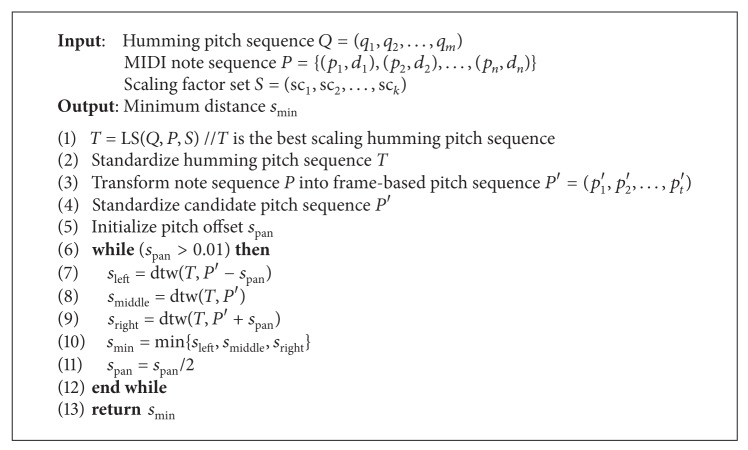
DTW-based match (*Q*, *P*, *S*).

**Algorithm 2:**
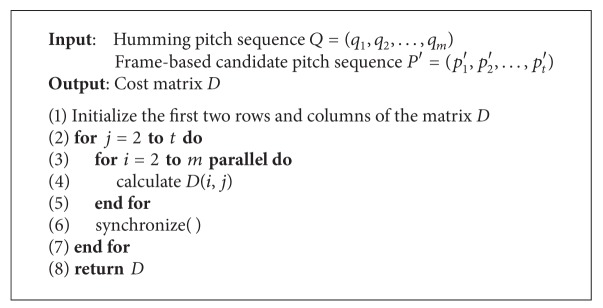
Parallel implementation for calculating the matrix *D* PDTW (*Q*, *P*).

**Algorithm 3:**
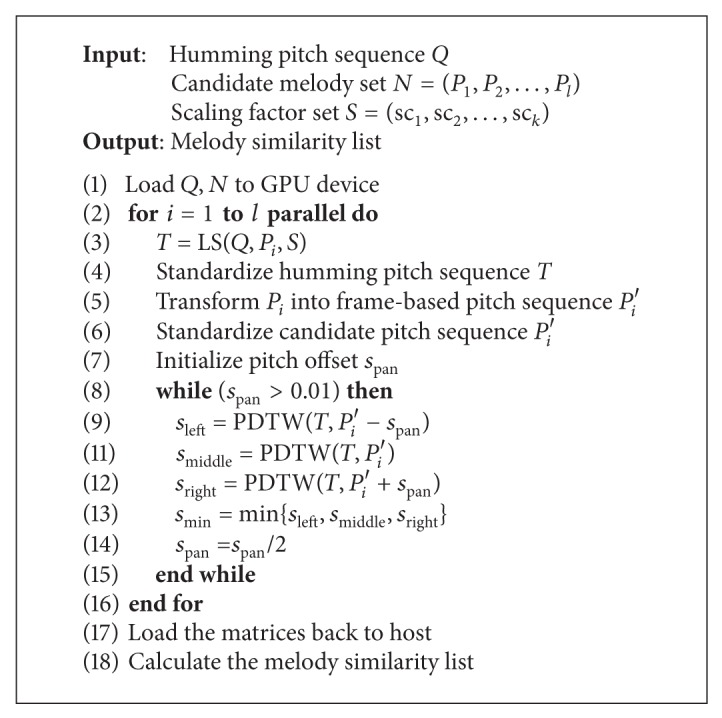
GPU-based parallel melody accurate matching.

**Table 1:** Evaluation environment.

	CPU (AthlonII X4)	GTX285	GTX480
Number of Core	1~4	240 (30SM)	480 (15SM)
SP clock	3.2 GHz	1.476 GHz	1.446 GHz
Complier	GCC 4.1.2	CUDA SDK 4.0	CUDA SDK 4.0

**Table 2:** Retrieval results for different methods.

Method	MRR	Top-1	Top-3	Top-5	Top-10
Serial version	0.821	0.762	0.809	0.905	0.952
Parallel version	0.813	0.725	0.807	0.925	0.957
